# Exploring the ITO/PET Extended-Gate Field-Effect Transistor (EGFET) for pH Sensing

**DOI:** 10.3390/s23208350

**Published:** 2023-10-10

**Authors:** Z. Mouffak, V. Adapala

**Affiliations:** Department of Electrical and Computer Engineering, California State University, Fresno, CA 93740, USA

**Keywords:** extended-gate field-effect transistor, EGFET, chemical sensors, pH sensing, ITO/PET

## Abstract

In this project we investigated the extended-gate field-effect transistor (EGFET) structure used with ITO (Indium Tin Oxide)/PET (Polyethylene Terephthalate) sensitive films acting as the extended-gate part of an EGFET obtained from a combination of FETs from the CD4007 chip. We tested the device as a pH sensor by immersing the ITO/PET electrode in several chemical solutions of acidic and basic nature, including hydrogen peroxide, acetic acid, sulfuric acid, and ammonium hydroxide, at different concentrations. Using a Tektronix 4200A sourcemeter, we plotted the current–voltage (I–V) characteristics for the different chemical solutions, and we established a correlation to the pH changes. Results from the plotted I–V characteristics show a great dependance of the drain current (I_D_) on solution concentration. Furthermore, we measured the pH of each of the used solutions, and we established a relationship between the drain current and the pH value. Our results show a consistent decrease in the current with an increase in the pH value, although with different rates depending on the solution. The device showed high voltage sensitivity at 0.23 V per pH unit when tested in sulfuric acid.

## 1. Introduction

Traditional pH meters have been limited by their bulky size, making them less convenient for many applications. However, researchers have delved into a promising alternative, ion-sensitive field-effect transistors (ISFETs) [1,2,3,4], which owe their existence to the development of the insulated-gate field-effect transistor (IGFET), the first solid-state device designed to detect ionic activity [5]. In ISFETs, the changes in drain current are due to the voltage disparity between the electrolyte solution and the silicon dioxide (SiO_2_) gate. In 1983, when an innovative spark ignited in the mind of Van der Spiegel [6], he laid the foundation for a novel invention: the extended-gate field-effect transistor, or EGFET, a progeny of the ISFET family [7]. EGFETs revolutionized the chemical sensing landscape by ingeniously integrating the gate terminal of a readily available metal-oxide-semiconductor field-effect transistor (MOSFET) with a specialized sensing film. This sensing film could be made from a range of materials, including Indium Tin Oxide (ITO) on Polyethylene Terephthalate (PET) [8], metals like gold, platinum, or silver [9], carbon-based materials [10], porous silicon [11], or polymers [12]. The EGFET’s operation relies on its sensitivity to alterations in the solution they are immersed in. EGFETs have found applications in diverse fields, particularly in pH measurement [13,14,15,16], enabling accurate determination of acidity and alkalinity. Beyond that, they have been employed in detecting urea [17], quantifying glucose levels [18], and even conducting DNA tests [19]. The working principle of EGFETs closely mirrors that of ISFETs. Instead of an electrolyte solution, the gate terminal is connected to a sensitive film, transforming it into an *extended gate* of the MOSFET. This film is then immersed in a solution, where it becomes exquisitely sensitive to changes in the solution’s properties. The sensor we built for this study consists of an ITO/PET sensing electrode dipped in the chemical solution along with a copper reference electrode. The sensing electrode is connected to the gate of the MOSFET while the reference electrode is directly connected to the Keithley 4200A sourcemeter where a bias voltage is applied (indirectly, a gate bias). The ITO EGFET sensor is simple to use and test, and very cheap due to the low cost of the chip (52 cents) and the ITO piece (~$1). There have been only a few articles on the ITO/PET EGFET sensor, and in all of them an acid and a base were mixed at different proportions to modify the pH. In this study we diluted different solutions to test the sensor, and we compared the sensor behavior. Our goal is to investigate the effectiveness of this low-cost sensor across a variety of chemicals.

## 2. Materials and Methods

**Figure 1:**
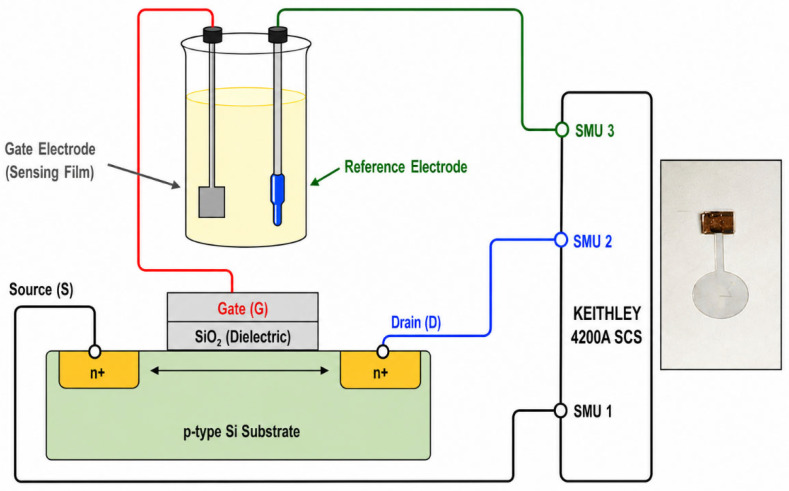
Proposed EGFET schematic diagram and the ITO/PET electrode used in this project. The ITO/PET electrode is immersed in the solution, along with a copper reference electrode.

We used Indium Tin Oxide (ITO)/Polyethylene Terephthalate (PET) as a sensing electrode to test the buffer solutions. An ITO/PET flexible sheet, from which we cut the electrode, was purchased from Adafruit (New York, NY, USA), and had a resistance of 50 Ω/square inch. The electrode was connected to the MOSFET gate terminal, then dipped in the solution. The reference electrode was used to apply the gate bias voltage. A second voltage was applied at the drain of the MOSFET, while the source was connected to ground. For each gate voltage applied, we varied the drain voltage and observed the drain current. Figure 1 shows the block diagram of the experimental setup for the project; we used a CD4007UB IC. This chip is used by most researchers who worked on EGFET due to its low cost and the possibility to connect several MOSFETs in parallel to form one MOSFET that can handle a higher current due to a larger resulting aspect ratio (W/L). In this IC there are 3 n-type and 3 p-type enhancement transistors. We connected the terminals of the IC containing n-type transistors using copper wires, as shown in the diagram in Figure 1. Buffer solutions of ammonium hydroxide (28–30% ammonia solution, 15.0 M) and hydrogen peroxide (30% solution, 9.8 M) were purchased from VWR International. Sulfuric acid (18.4 M) was purchased from Fisher Scientific. We used commercial acetic acid (vinegar, 5%, ~8 M). Distilled water was mixed with varying concentrations of hydrogen peroxide, sulfuric acid, ammonium peroxide, and acetic acid to prepare the test solutions.

Once all the n-type transistors in the IC were wired up on the breadboard, we tested the setup with the Keithley 4200A SCS (Tektronix, Inc., Beaverton, OR, USA) parameter analyzer by running an I–V characteristic test. Before we ran our project setup, the I–V characteristics of CD4007UB were tested with one transistor and with the combination of the 3 n-type transistors. As expected, we observed that the combination of n-type transistors drew more current compared to single transistor. For this reason, we combined the n-type transistors throughout the experiment, as shown in Figure 1.

When an n-type MOSFET’s gate terminal is connected to an ITO/PET electrode film, we obtain an EGFET sensor. This is a single ITO/PET electrode that we designed in different shapes. To avoid the mechanical strain on the sensing film that occurs when using scissors or a cutter to cut it, we used laser cutting [20]. A round-shaped laser-cut electrode yielded maximum current, so we adopted that shape. In the EGFET device, the round-shaped ITO/PET electrode was connected to the gate of the MOSFET. The experiment relied on a reference copper electrode that we immersed in the chemical solution. When the transistor is activated with a constant voltage of the reference electrode, measurement noise is reduced. The ITO/PET electrode was only immersed in the solution for enough time to run the I–V characteristic test. The total exposure time was 30 s.

The voltage V_DS_ varied from 0 to 5 volts, whereas we used voltages of 2, 3, 4, and 5 volts for the gate. We connected the ITO/PET electrode to the gate terminal and dipped it into the solution. Distilled water was mixed with varying concentrations of hydrogen peroxide, sulfuric acid, ammonium peroxide, and acetic acid to prepare the test solutions.

## 3. Results and Discussion

**Figure 2:**
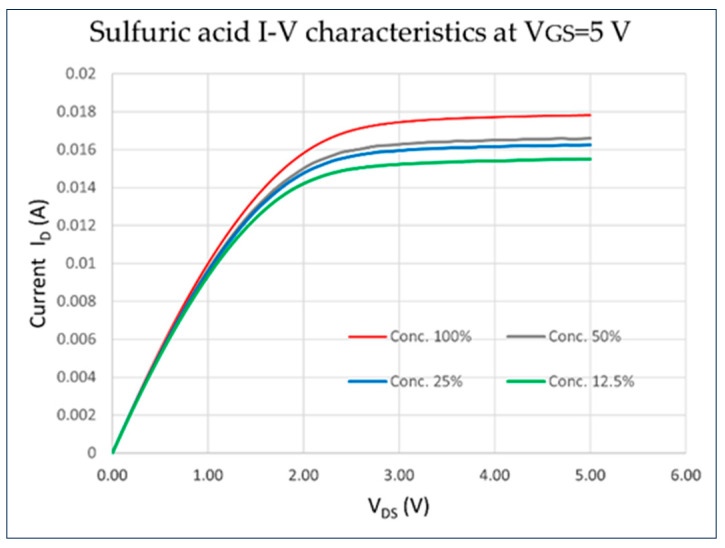
Sulfuric acid I–V characteristics for different concentrations for applied gate voltage V_GS_ = 5 V. There is a higher current with a higher acid concentration (lower pH). This experiment was run for the three other solutions and gave similar results.

Figure 2 shows the I–V characteristics for a gate bias V_GS_ = 5 V of the EGFET device used in sulfuric acid solutions with different concentrations of 12.5%, 25%, 50%, and 100% in distilled water. The goal here was to test the sensitivity of the EGFET to various concentrations of acid. These solutions had pH values of 0.90, 0.45, 0.42, and 0.36 respectively. We were able to observe an increase in drain current as the concentration increases. To properly estimate the pH sensing of this device, we ran a different set of experiments; we still used sulfuric acid, but used high dilutions to reach a wider range of pH values. We used a commercial pH meter (Tuecota 0.01 High Accuracy Digital pH Water Tester, Measuring Range 0–14) to measure the pH of the solutions.

**Figure 3:**
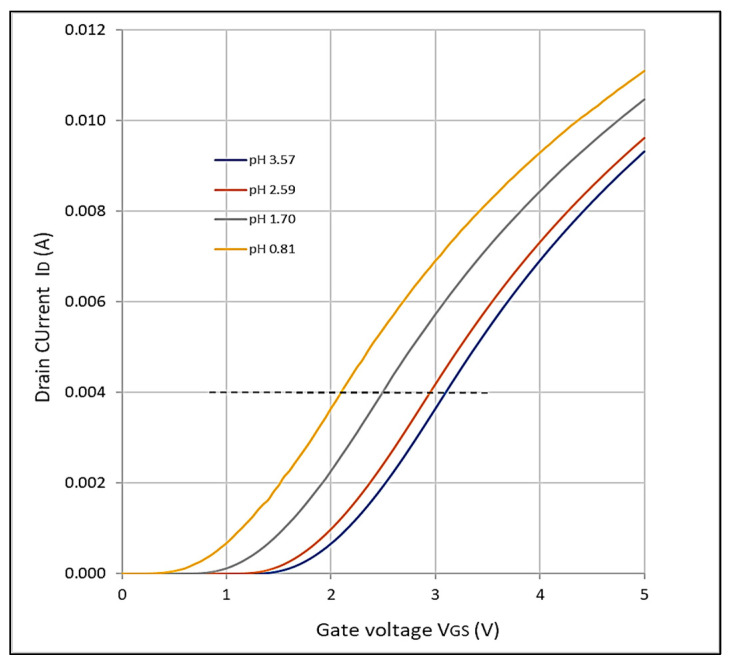
I_D_ vs. V_GS_ characteristics for sulfuric acid aqueous solutions with concentrations of 10%, 1%, 0.1%, and 0.01% and corresponding pH values 0.8, 1.70, 2.59, and 3.57. The higher the concentration of acid, the lower the EGFET threshold voltage. The horizontal line shows the values of voltage used to determine the sensor voltage sensitivity.

We used concentrations of sulfuric acid in distilled water at 10%, 0.1%, 0.01%, and 0.001%, and we measured the corresponding pH values to be 0.81, 1.70, 2.59, and 3.57, respectively, which confirms the fact that the pH of an acidic solution increases by 1 when diluted by a factor of 10 (errors are due to pH-meter measurement errors and small titration human reading errors). We ran measurements of the drain current as a function of the gate voltage. Characteristics are plotted in Figure 3, where we can see the threshold voltage of the solutions described above. We can observe that the smaller the pH, the smaller the threshold voltage, i.e., currents are higher with higher concentrations of acid for the same gate voltage.

To determine the device sensitivity to pH in terms of voltage, we drew a horizontal line to look at the voltages required for each solution to yield the same drain current. Here we used a drain current of 4 mA. The voltages collected were 2.10 V, 2.50 V, 2.95 V, and 3.10 V for solutions with pHs 0.81, 1.70, 2.59, and 3.57, respectively. This translates to an average of ~0.23 V per unit pH, which is remarkable.

**Figure 4:**
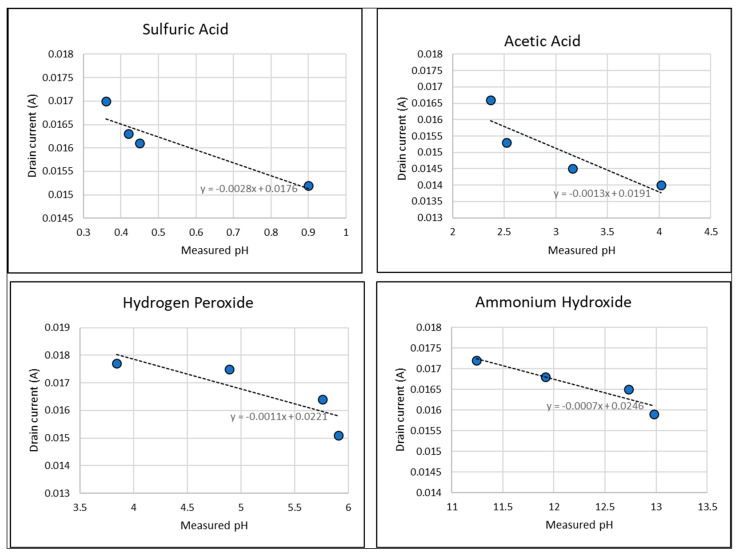
Drain current at V_GS_ = 5 V and V_DS_ = 4 V as a function of the measured pH of four solutions: sulfuric acid, acetic acid, hydrogen peroxide, and ammonium hydroxide. We see a consistent linear decrease in the current as the pH increases.

In this study, we were interested in both pH sensing and specific solution concentration detection, so we tested and compared four chemical solutions: sulfuric acid, acetic acid, hydrogen peroxide, and ammonium hydroxide. Before each I–V characteristic run, we measured the pH of our chemical solution. Table 1 shows values of measured pH values of the solutions and the corresponding drain current measured when the gate voltage bias was V_GS_ = 5 V and the drain voltage was V_DS_ = 4 V. Figure 4 shows the plots of the drain current measured from the EGFET as a function of the measured pH. As expected, there is a consistent decrease in current as the value of the pH increases in each solution. As the pH of the solution increases, the surface charge of the sensing layer becomes more negative due to the dissociation of functional groups on the layer. This results in a shift in the I–V characteristics of the EGFET sensor.

The results obtained from our experiments reveal a clear and consistent trend in the behavior of the ITO/PET EGFET sensing device with varying solution concentrations and pH levels. Notably, we observed an increase in the drain current when the solution was acidic, and conversely, a decrease in drain current when a basic solution was used. This observation aligns perfectly with the fundamental principles of pH and its relationship with the concentration of hydrogen ions (H^+^) in solution. In the case of acidic solutions, the increase in drain current can be attributed to the higher concentration of H^+^ ions. As the concentration of H^+^ ions increases in an acidic solution, the solution’s pH level decreases (having a higher acid concentration). This increase in H^+^ ions leads to a higher positive charge at the gate electrode–electrolyte interface, which helps to have more inversion in the channel area of the EGFET, and enhances the channel’s conduction. Consequently, this results in an increase in the drain current. On the other hand, when a basic solution was introduced, characterized by a higher pH level due to a lower concentration of H^+^ ions, we observed a decrease in drain current. This can be attributed to a weaker positive charge at the gate electrode–electrolyte interface. This weakened positive charge allows for a less conducting EGFET channel, which in turn leads to a decrease in the drain current. As Figure 2 shows, the higher the concentration of sulfuric acid—and of course lower pH—the higher the current. We ran the I–V characteristics of the other three solutions as well (acetic acid, hydrogen peroxide, and ammonium hydroxide), and we noted similar results. For ammonium hydroxide, a dilution of the solution resulted in higher current due to lower pH.

Our plots of the drain current versus pH values show a consistent decrease in current with an increase in the pH of a given solution. However, the solutions here show slightly different effects on current. For instance, sulfuric acid at a concentration of 12.5% draws a drain current of 15.2 mA for a pH of 0.9, while a solution with a much higher pH of 12.98 (diluted ammonium hydroxide) draws a higher drain current of 17.2 mA. Similar comparisons can be made between results from hydrogen peroxide solutions and those of ammonium hydroxide. Furthermore, the variation of current with the pH depends on the solution. Sulfuric acid shows the highest change with pH at 28 mA per pH unit, while acetic acid, hydrogen peroxide, and ammonium hydroxide show a current change of 13, 11, and 7 mA/pH unit, respectively. It is worth mentioning that sulfuric acid solutions had a much higher temperature that we did not account for, and as the temperature of a solution increases, its electrical conductivity increases due to the greater kinetic energy of ions in the solution. Consequently, more ions are available for electrical conduction, resulting in higher conductivity, which could explain the higher current rate per pH unit.

These results demonstrate that the ITO/PET EGFET sensing device exhibits a strong correlation between drain current, the concentration of the solution, and its pH. This correlation reaffirms the device’s remarkable potential as a chemical sensor, specifically an effective pH sensor, which is in line with a previously published work [20]. However, pH sensing sensitivity changes from one solution to another. The ability to accurately detect and quantify pH levels across a range of solutions positions the ITO/PET EGFET as a valuable tool in various applications that can be further explored, including environmental monitoring, biomedical research, and industrial processes.

## 4. Conclusions

In conclusion, our experiments of the EGFET sensor using an ITO/PET electrode yielded electrical characteristics reaffirming its potential as an effective pH sensor. The I–V characteristics unveiled a linear trend in response to diverse chemical solutions. There is a notable decrease in drain current when diluting acidic solutions, contrasting with an increase in current when diluting alkaline solutions. Our I_D_–V_GS_ electrical characteristics show that the EGFET threshold voltage increases as the pH value increases, with a very high average voltage sensitivity of ~0.23 V per pH unit. We also note that the rate of change in drain current relative to pH depends on the solution itself, since the rate of change in drain current relative to pH slightly depends on the solution used.

## Figures and Tables

**Table 1:** Drain current and measured pH values of different chemical solutions at different dilution rates.

Scheme	Measured pH	Measured I_D_ (A)
Acetic acid (diluted at 12.5%)	4.02	0.0166
Acetic acid (diluted at 25%)	3.16	0.0153
Acetic acid (diluted at 50%)	2.52	0.0145
Acetic acid (full 6%)	2.37	0.014
Sulfuric acid (diluted at 12.5%)	0.9	0.0178
Sulfuric acid (diluted at 25%)	0.45	0.0163
Sulfuric acid (diluted at 50%)	0.42	0.0161
Sulfuric acid 100% (full)	0.36	0.0152
Hydrogen peroxide (diluted at 12.5%)	5.91	0.0177
Hydrogen peroxide (diluted at 25%)	5.76	0.0175
Hydrogen peroxide (diluted at 50%)	4.89	0.0164
Hydrogen peroxide 100% (full)	3.84	0.0151
Ammonium hydroxide (diluted at 12.5%)	11.24	0.0158
Ammonium hydroxide (diluted at 25%)	11.92	0.0165
Ammonium hydroxide (diluted at 50%)	12.73	0.0168
Ammonium hydroxide 100% (full)	12.98	0.0172
