# Correction: Mouffak, Z.; Adapala, V. Exploring the ITO/PET Extended-Gate Field-Effect Transistor (EGFET) for pH Sensing. *Sensors* 2023, *23*, 8350

**DOI:** 10.3390/s26113568

**Published:** 2026-06-04

**Authors:** Z. Mouffak, V. Adapala

**Affiliations:** Department of Electrical and Computer Engineering, California State University, Fresno, CA 93740, USA

**Figure Correction**

In the original publication [1], there was a mistake in the published version of Figure 1. The authors have now provided a redrawn Figure 1 to replace the original one. The corrected Figure 1 appears below.

The authors state that the scientific conclusions are unaffected. This correction was approved by the Academic Editor. The original publication has also been updated.

## Figures and Tables

**Figure 1:**
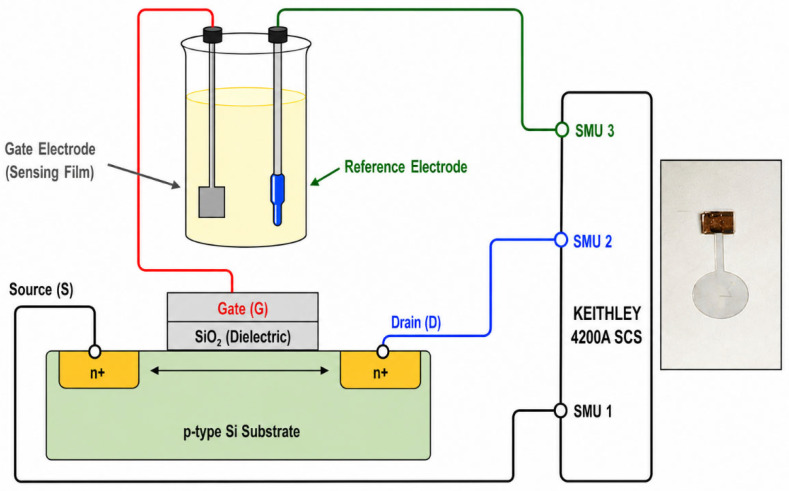
Proposed EGFET schematic diagram and the ITO/PET electrode used in this project. The ITO/PET electrode is immersed in the solution, along with a copper reference electrode.
